# Handcrafted and Deep Learning-Based Radiomic Models Can Distinguish GBM from Brain Metastasis

**DOI:** 10.1155/2021/5518717

**Published:** 2021-06-03

**Authors:** Zhiyuan Liu, Zekun Jiang, Li Meng, Jun Yang, Ying Liu, Yingying Zhang, Haiqin Peng, Jiahui Li, Gang Xiao, Zijian Zhang, Rongrong Zhou

**Affiliations:** ^1^Department of Oncology, Xiangya Hospital, Central South University, Changsha 410008, China; ^2^Xiangya Lung Cancer Center, Xiangya Hospital, Central South University, Changsha 410008, China; ^3^Shandong Key Laboratory of Medical Physics and Image Processing, Shandong Institute of Industrial Technology for Health Sciences and Precision Medicine, School of Physics and Electronics, Shandong Normal University, Jinan 250358, Shandong, China; ^4^Department of Radiology, Xiangya Hospital, Central South University, Changsha 410008, China; ^5^Department of Radiology, The Third Affiliated Hospital of Kunming Medical University, Yunnan Cancer Hospita and Center, Kunming 650100, Yunnan, China; ^6^Department of Radiation Oncology, Nanfang Hospital, Southern Medical University, Guangzhou 510515, China

## Abstract

**Objective:**

The purpose of this study was to investigate the feasibility of applying handcrafted radiomics (HCR) and deep learning-based radiomics (DLR) for the accurate preoperative classification of glioblastoma (GBM) and solitary brain metastasis (BM).

**Methods:**

A retrospective analysis of the magnetic resonance imaging (MRI) data of 140 patients (110 in the training dataset and 30 in the test dataset) with GBM and 128 patients (98 in the training dataset and 30 in the test dataset) with BM confirmed by surgical pathology was performed. The regions of interest (ROIs) on T1-weighted imaging (T1WI), T2-weighted imaging (T2WI), and contrast-enhanced T1WI (T1CE) were drawn manually, and then, HCR and DLR analyses were performed. On this basis, different machine learning algorithms were implemented and compared to find the optimal modeling method. The final classifiers were identified and validated for different MRI modalities using HCR features and HCR + DLR features. By analyzing the receiver operating characteristic (ROC) curve, the area under the curve (AUC), accuracy, sensitivity, and specificity were calculated to evaluate the predictive efficacy of different methods.

**Results:**

In multiclassifier modeling, random forest modeling showed the best distinguishing performance among all MRI modalities. HCR models already showed good results for distinguishing between the two types of brain tumors in the test dataset (T1WI, AUC = 0.86; T2WI, AUC = 0.76; T1CE, AUC = 0.93). By adding DLR features, all AUCs showed significant improvement (T1WI, AUC = 0.87; T2WI, AUC = 0.80; T1CE, AUC = 0.97; *p* < 0.05). The T1CE-based radiomic model showed the best classification performance (AUC = 0.99 in the training dataset and AUC = 0.97 in the test dataset), surpassing the other MRI modalities (*p* < 0.05). The multimodality radiomic model also showed robust performance (AUC = 1 in the training dataset and AUC = 0.84 in the test dataset).

**Conclusion:**

Machine learning models using MRI radiomic features can help distinguish GBM from BM effectively, especially the combination of HCR and DLR features.

## 1. Introduction

Brain metastases (BMs) are the most common tumors of the central nervous system (CNS), with an incidence of approximately 7–14 per 100,000 population [1], whereas glioblastoma (GBM) has an incidence rate of 3.22 per 100,000 population and is the most common malignant primary brain tumor in adults [2]. According to the World Health Organization (WHO) classification of CNS tumors, gliomas can be categorized as grades I–IV based on histological characteristics; GBM is categorized as a WHO grade IV glioma, accounting for the majority of gliomas [3]. According to the statistics of the Central Brain Tumor Registry of the United States (CBTRUS), the incidence of BM and GBM increases each year [2]. Patients with BM have a median survival of 4–6 months [4], while the median survival of GBM patients is 14 months [5], despite surgery, chemotherapy, and radiation therapy. At present, the standard treatment for BM is stereotactic radiotherapy, while the most effective therapeutic method for GBM is surgery. Therefore, an accurate preoperative diagnosis is of significance for surgical planning, determining the extent of resection [6], evaluating the need for neoadjuvant therapy, defining the radiation therapy field, and counseling patients and their families [7].

It is generally accepted that magnetic resonance imaging (MRI) is an important modality for evaluating brain tumors. In patients with a history of systemic cancer and multiple lesions, the differentiation of BM from GBM may be easily achieved using conventional MRI. However, single metastases were estimated to occur in more than 25% of cases of BM [8]. Additionally, systemic malignancy was present in approximately 3% of high-grade glioma cases [9], and multifocal lesions accounted for up to 20% of GBM cases in some reports [10]. Furthermore, as both BM and GBM can present with contrast-enhancing and necrotic areas, they often present a similar anatomical appearance on MRI [8]. Therefore, the use of conventional MRI in differentiating between single BMs and GBM lesions is limited.

Generally, BM and GBM are distinguished by some observable MRI features. Lesions that show nodular or ring-shaped inhomogeneous contrast enhancement and hypointense signals on T1-weighted imaging (T1WI) and hyperintense signals on T2-weighted imaging (T2WI) are visible on conventional MRI for both diseases. Compared with GBM lesions, most BM lesions are multifocal, have relatively clear boundaries, and are surrounded by severe edema. Moreover, BM lesions have smaller volumes, smaller areas of necrosis and cystic degeneration, and less enhancement than GBM lesions. Nevertheless, these methods, with limited sensitivity and specificity, are susceptible to individual subjectivity, resulting in differences among radiologists. However, the use of multiple MRI techniques is expected to significantly improve the diagnostic results. Several studies have differentiated between GBM and BM based on multiparametric MRI data, including data from advanced imaging methods such as diffusion [11, 12], perfusion [13, 14], and MR spectroscopy [15, 16]. Askaner et al. made a differential diagnosis by statistically analyzing the relative cerebral blood volume of the solid tumor area, peritumor area, and adjacent area on perfusion-weighted imaging (PWI) [17]. The apparent diffusion coefficient was used to compare and distinguish both diseases on diffusion-weighted imaging (DWI) by Lee et al. [18]. Nevertheless, there is no widely held standard to distinguish between GBM and BM, except histopathological evaluation. Moreover, these advanced imaging methods are not readymade tools in most radiology departments because they are relatively time consuming, which limits their clinical promotion.

While histopathological evaluation is currently the gold standard for brain tumor diagnosis [19], there is a growing body of evidence that the combination of quantitative imaging and machine learning algorithms can help with the noninvasive differentiation of brain neoplasms based on pretreatment MRI [20]. Radiomics is an emerging field that aims to utilize the full potential of medical imaging by extracting a large number of quantitative features, including tumor intensity, shape, and texture [21]. Radiomics has recently emerged as a powerful methodology to quantify the characteristics of tumors and mine more biological information in a noninvasive manner [22]. Many studies have demonstrated that distinct tumor types in many organs can be quantified by radiomic analysis, and the results of radiomics can be used as imaging biomarkers to support clinical decision making [23–25]. Radiomics can also reveal novel characteristics of brain tumors, as demonstrated by a recent study. Radiomic analysis has been shown to improve diagnosis, prognosis, and decision making in the treatment of patients over standard radiological assessment [26]. Machine learning models can combine a large number of variables of different data types in a single model, thereby maximizing the efficacy of prediction testing. Machine learning technology has been widely used to diagnose various types of tumors.

Previous studies have shown that radiomics offers important advantages in the assessment of the underlying tumor pathophysiology and improves the ability to distinguish between tumors [27–29]. Although multiple classifiers have been found in many previous radiomic studies, the main purpose was to find the best classifier and not validate the additional radiomic features. Because different radiomic feature groups may have their own advantages or disadvantages due to the feature extraction method, we hypothesized that the combined use of handcrafted radiomics (HCR) and deep learning-based radiomics (DLR) might provide extra benefits. To our knowledge, there have been no reports validating the potential of the combination of HCR and DLR for the classification of GBM and solitary BM lesions. Thus, the present study sought to differentiate single BM from GBM lesions by combining high-dimensional radiomic features based on conventional MRI and machine learning technologies.

## 2. Materials and Methods

**Figure 1:**
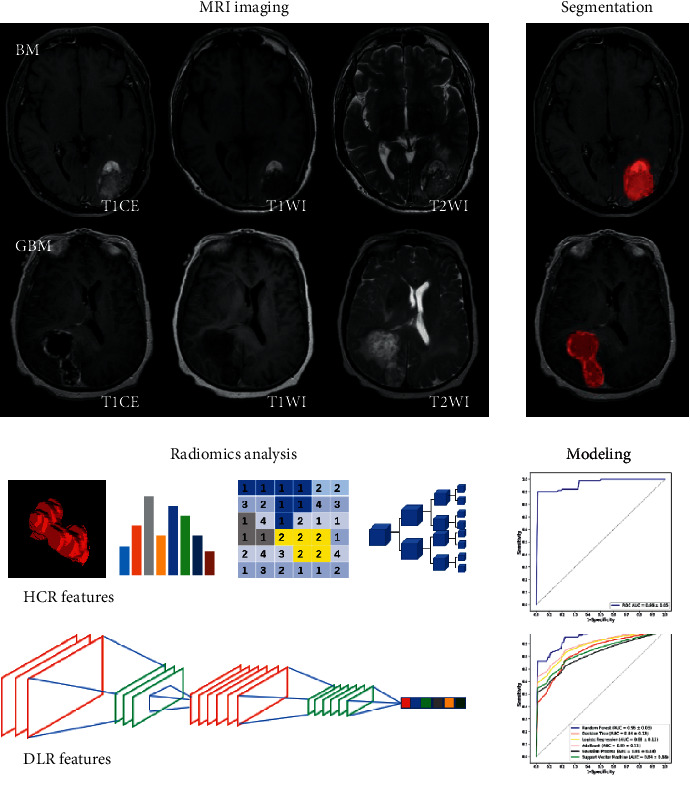
Study workflow overview.

The study was approved by the Institutional Review Board (IRB) of Xiangya Hospital. According to the relevant guidelines and regulations of the retrospective study, the requirement for informed consent was waived. The study workflow overview is shown in Figure 1.

### 2.1. Patient Data

This was a retrospective analysis of 268 patients with GBM and BM from Xiangya Hospital, Yunnan Cancer Hospital, and Nanfang Hospital, proven pathologically between January 1, 2010, and December 31, 2018. Preoperative routine plain and enhanced MRI scans were required for patients to be eligible for inclusion in the study. Patients who had a treatment history (including surgery and radiotherapy) related to GBM or BM were excluded. The 268 patients were separated into two groups: 208 (98 BM and 110 GBM) patients from Xiangya Hospital were included in the training dataset and 60 (30 BM and 30 GBM) patients from Yunnan Cancer Hospital and Nanfang Hospital were included in the test dataset. The diagnoses of all patients were confirmed histologically on the basis of the 2016 WHO classification system.

### 2.2. MRI Protocol

All MRI examinations were conducted in the radiology department of Xiangya Hospital, Yunnan Cancer Hospital, and Nanfang Hospital with a 3.0-T MRI system. High-quality MRI images were obtained using the following protocols: axial T1WI: layer thickness = 5 mm, layer spacing = 1.5 mm, matrix = 320×256, and field of view (FOV) = 24 × 24 cm; axial T2WI: layer thickness = 5 mm, layer spacing = 1.5 mm, matrix = 384× 384, and FOV = 24 × 24 cm; and axial contrast-enhanced T1WI (T1CE): layer thickness = 5 mm, layer spacing = 1.5 mm, matrix = 320 × 256, and FOV = 24 × 24 cm. All MRI images were retrieved from the picture archiving and communication system for further image feature extraction.

### 2.3. Region of Interest (ROI) Preprocessing

Through noise reduction, offset field correction, and strict object internal registration, we preprocessed each image using the public software package FSL. Histogram matching was performed such that the intensity levels between various objects were comparable. All images were assessed by two neuroradiologists (each with 5–10 years of work experience) independently. The ROIs of the entire tumor on T1WI, T2WI, and T1CE images were created manually around the enhanced part of the tumor layer by layer using ITK-SNAP software [30]; areas of macroscopic necrosis, cystic changes, and edema were avoided. A third senior neuroradiologist (with 15 years of work experience) reexamined the images and made a final diagnosis when there was an inconsistency between the two neuroradiologists.

### 2.4. HCR Feature Extraction

Based on the segmentation of ROIs, we performed HCR feature extraction using PyRadiomics [31]. The high-throughput HCR features included 16 shape-based features, 18 histogram features, 20 gray level co-occurrence matrix (GLCM) features, 14 gray level dependence matrix (GLDM) features, 16 gray level run length matrix (GLRLM) features, 16 gray level size zone matrix (GLSZM) features, 5 neighboring gray tone difference matrix (NGTDM) features, 728 wavelet features, and 273 Laplacian of Gaussian (LoG) features. The details of these features are available in PyRadiomics documentation (https://pyradiomics.readthedocs.io/en/latest/index.html). Finally, 1106 HCR features were extracted from each MRI ROI, and 3318 HCR features were extracted from each patient.

### 2.5. DLR Feature Extraction

A convolutional neural network (CNN) was also used to extract and summarize MRI features. Here, we used a pretrained ResNet transferred from ImageNet, which was built by Keras. The last fully convolutional layer was reset, and each ResNet could extract 1000 features from each MRI modality.

### 2.6. Feature Selection and Machine Learning Modeling

**Figure 2:**
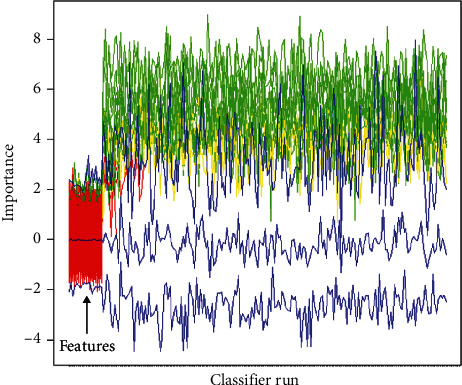
The Boruta selection of HCR + DLR features of T1CE data. Green and yellow indicate high-importance features, and blue and red indicate low-importance features.

Based on the HCR and DLR features, the random forest- (RF-) based Boruta algorithm was applied to select the optimal feature groups. Boruta is a wrapper algorithm that performs robust, statistically grounded feature selection for all relevant features [32]. By comparing the importance of the original attribute with the importance of its randomized copies [33], all relevant features were selected for subsequent modeling. One RF-Boruta selection example is shown in Figure 2. Then, for the selected HCR features and HCR + DLR features of the three MRI modalities, a total of 6 radiomic feature groups were advanced to the next modeling step. To find the optimal modeling method, six different machine learning algorithms were adopted to build the classifiers for the 6 feature groups. RF, decision tree, logistic regression, AdaBoost, Gaussian processing, and support vector machine were implemented using 10-fold cross-validation of the training dataset in turn. Receiver operating characteristic (ROC) curve analysis was performed, and the area under the curve (AUC) of each model was obtained to evaluate the predictive performance.

### 2.7. Model Establishment and Validation

After finding the best modeling method, each classifier was established and validated in the training dataset and tested in the test dataset. The HCR models of the T1CE, T1WI, T2WI, and multimodality features were built and compared; then, DLR features were added, and the above process was repeated. Through comparison of the ROC curves and AUC values, the optimal MRI modality and machine learning model were identified.

### 2.8. Statistical Analysis

All machine learning algorithms were implemented using scikit-learn and Keras libraries, and statistical analysis was carried out using SPSS (version 26, IBM Corporation, Armonk, NY, USA). Independent *t*-tests were used to assess differences in continuous variables between the patient groups, and Fisher's exact tests were used to assess noncontinuous variables. The DeLong test was performed to compare ROC curves. A *P* value < 0.05 was considered statistically significant.

## 3. Results

### 3.1. Patient Characteristics

The clinical characteristics of the patients in the training and test cohorts are given in Table 1. There was no significant difference in age or sex between patients with GBM and BM in the two cohorts.

### 3.2. Radiomics Features and Machine Learning Modeling

**Figure 3:**
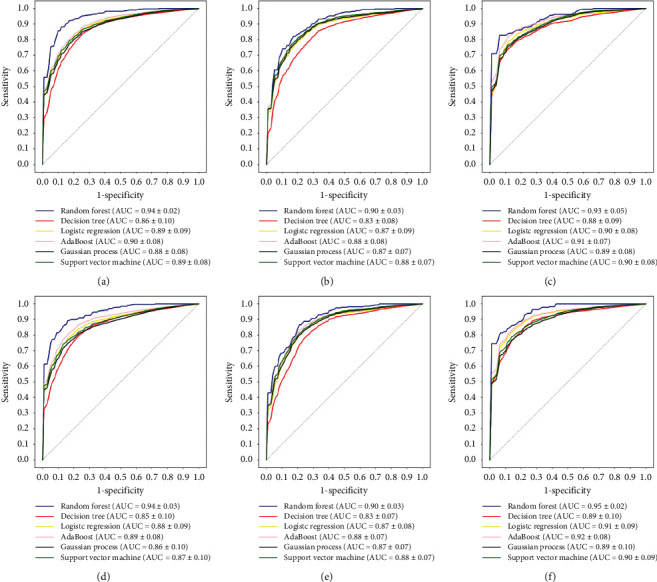
Performance of different machine learning modeling methods in 6 feature groups. (a)–(c) ROC curves of the HCR models based on T1CE, T1WI, and T2WI data, respectively. (d)–(f) ROC curves of the HCR + DLR models based on T1CE, T1WI, and T2WI data, respectively.

A total of 1106 HCR features and 1000 DLR features were extracted for each case from T1WI, T2WI, and T1CE data. By Boruta selection, the relevant features were selected; the details of each feature group are given in Supplementary Material 1. For all 6 feature groups, the RF models all showed better performance than the other machine learning algorithms (*p* < 0.05) (Figure 3).

**Figure 4:**
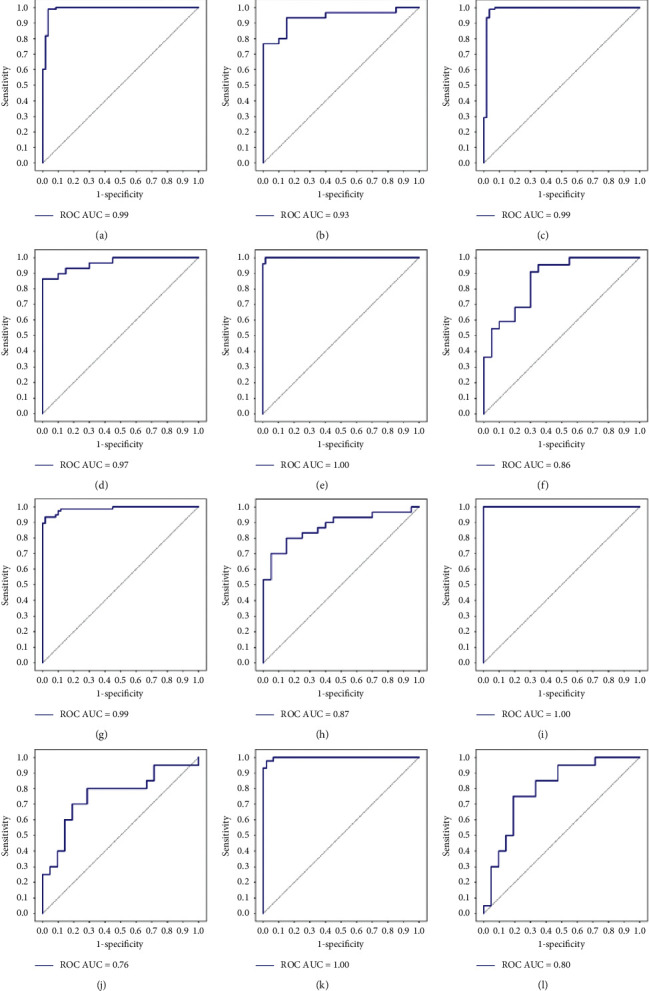
Diagnostic performance of 6 feature groups. (a)-(b) ROC curves of the HCR model based on T1CE data in the training dataset and test dataset, respectively. (c)-(d) HCR + DLR models of T1CE data in the training and test datasets. (e)–(h) HCR and HCR + DLR models of T1WI data in the training dataset and test dataset. (i)–(l) Details of T2WI data.

With only HCR features, T1CE showed the best distinguishing performance (AUC = 0.99, in the training dataset; AUC = 0.93, in the test dataset), which was significantly better than that of T1WI and T2WI (*p* < 0.05). Adding DLR features, T1CE showed significant improvement (AUC = 0.99, in the training dataset; AUC = 0.97, in the test dataset; *p* < 0.05) and remained the best model. T1WI (AUC = 0.99, in the training dataset; AUC = 0.87, in the test dataset; *p* < 0.05) and T2WI (AUC = 1.00, in the training dataset; AUC = 0.80, in the test dataset; *p* < 0.05) also showed significant improvement. All details are shown in Figure 4. Supplementary Material 2 shows the robust performance in the training dataset using 10-fold cross-validation.

**Figure 5:**
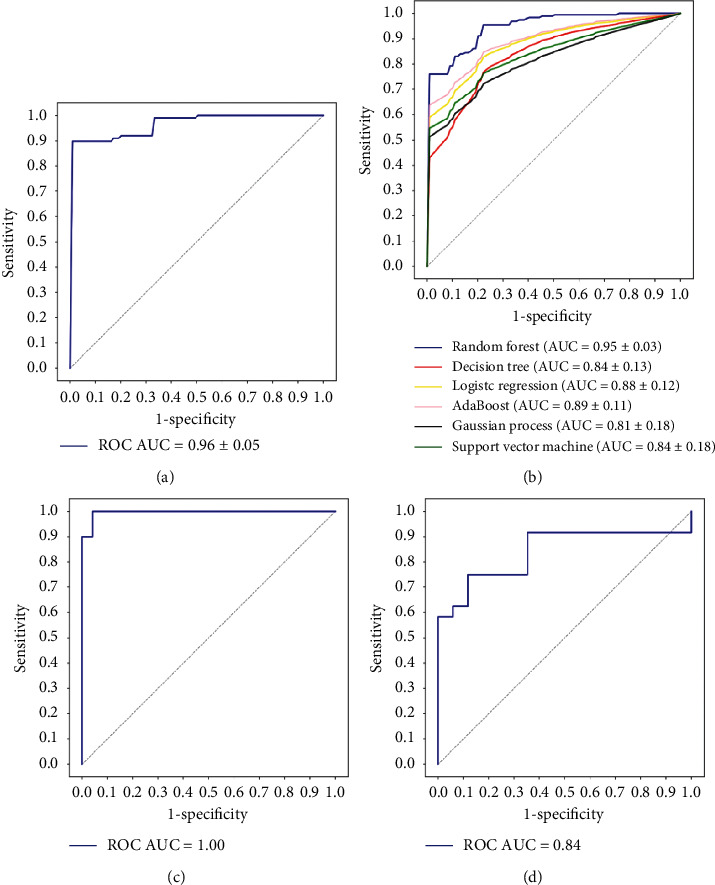
ROC curves of the multimodality radiomic model. (a) Results of 10-fold cross-validation in the training dataset. (b) Comparison of different machine learning models. (c) Final multimodality model in the training dataset. (d) Final multimodality model in the test dataset.

Generally, multimodality radiomic models show better diagnostic performance than single-modality models. In our study, the multimodality model showed an AUC of 1.00 in the training dataset and an AUC of 0.84 in the test dataset, which were lower than those of the T1CE- and T1WI-based radiomic models (*p* < 0.05).  Table 2 and Figure 5 show a comparison of the performance of the multimodality and single-modality models.

## 4. Discussion

The accurate classification of GBM and single BM lesions is a challenging clinical problem. Here, we provide a radiomic method based on HCR combined with DLR that showed potential for clinical application.

In this study, to overcome the disadvantages of current studies, we used different machine learning methods to preoperatively classify GBM and BM using a series of radiomic features. We evaluated tumor features throughout the tumor area rather than performing a largest cross-sectional analysis, since the analysis of three-dimensional tumor features may provide more diverse internal information than that of two-dimensional features. The majority of these features, such as wavelet, second-order, and some first-order statistical features, cannot be identified and quantified by the human eye, which highlights the advantage of using automatic methods and extracting high-order statistical features to assist in radiological assessment and clinical decision making. Furthermore, we carried out 10-fold cross-validation and multicenter verification, which enabled us to reduce regional deviation and improve the universality of the approach. The predictive models were built using HCR and DLR analysis. Based on HCR features, six different machine learning algorithms were implemented, including RF, decision tree, logistic regression, AdaBoost, Gaussian processing, and support vector machine. The RF classifier showed the best performance (AUC = 0.93). The combined radiomic model, based on the optimal HCR features and DLR features, showed the best overall performance, with an AUC of 0.99 in the training set and 0.97 in the test set. However, we should note that the differences in performance among the different models were not large enough to select one optimal model, specifically considering that the investigated models seemed to perform quite comparably and that the variance in AUC might be partially attributed to the small sample size. Therefore, our results can only be regarded to support our hypothesis and need to be verified in future studies.

ResNet was proposed in 2015 as a CNN to solve the problem of deep networks, such as the vanishing gradient [34]. Residual neural networks reduce the complexity by skipping the connection that skips training from a few layers and connects directly to the output. Skipping effectively simplifies the network, using fewer layers in the initial training stages, makes it easier to optimize, and provides additional accuracy from a considerably increased depth, which makes it possible to train up to hundreds or even thousands of layers and still achieve compelling performance. Taking advantage of its image classification capability, ResNet is a powerful potential tool for clinical imaging diagnosis. Some other deep learning methods have also been developed to classify and detect brain tumors [35, 36], all of which showed the clinical potential of artificial intelligence technology.

Generally, the potential of multimodality MRI for application may be better than that of single-modality MRI, but there are some cases in which its performance can be limited. In this study, the simple combination of selected features from images obtained by different MRI modalities may be one cause of limited performance; we know that more heterogeneous data do not necessarily provide more valuable information. Moreover, some studies have shown that single-modality models may have predictive potential better than or equal to that of multimodality models [37, 38]. We think that detrimental features usually decrease the overall performance of the model.

There are some limitations to this study that should be addressed. First, this was a retrospective analysis of a small sample. Second, the ratio of BM to GBM lesions and the variety of BM sources were not representative of the general population, which could exhibit different characteristics. Thus, a larger prospective study is needed for verification. Third, in this study, a manual method was used to segment ROIs. Although manual segmentation is usually better than automatic segmentation and two researchers participated in the segmentation process, segmentation errors still occur. Fourth, we did not compare the performance of the machine learning model with that of a human reader.

## 5. Conclusions

In conclusion, our study suggests a radiomic machine learning method for distinguishing BM from GBM lesions preoperatively with favorable predictive accuracy and stability. In addition, this combination could yield valuable insight into tumor progression, which could also facilitate the implementation of a personalized approach in tumor management. However, the results of our study need to be used with caution; we only validated the feasibility of applying HCR and DLR for the accurate preoperative classification of GBM and solitary BM lesions.

## Figures and Tables

**Table 1:** Cohort characteristics of 268 patients with BM and GBM.

Characteristics	Groups	Full (*n* = 268)	Training (*n* = 208)	Testing (*n* = 60)	*P* value
Age (years)	Mean±	55.48	55.63	54.96	0.74
	SD	9.18	9.36	8.68	

Gender	Male	170	138	33	0.22
	Female	98	70	27	

Tumor types	BM	128	98	30	
	GBM	140	110	30	

**Table 2:** The performance comparison of multimodality and single-modality models.

Models	Training AUC	Cross-validation mean AUC	Test AUC	Accuracy	Sensitivity	Specificity
T1CE-HCR	0.99	0.94	0.93	0.82	0.70	0.93
T1CE-HCR+DLR	**0.99**	**0.95**	**0.97**	**0.85**	**0.84**	**0.93**
T1WI-HCR	1.00	0.90	0.86	0.71	0.75	0.78
T1WI-HCR+DLR	0.99	0.91	0.87	0.76	0.75	0.83
T2WI-HCR	1.00	0.95	0.76	0.73	0.85	0.66
T2WI-HCR+DLR	1.00	0.96	0.80	0.78	0.80	0.83
Multimodality-HCR	1.00	0.92	0.81	0.71	0.70	0.82
Multimodality-HCR + DLR	1.00	0.96	0.84	0.75	0.71	0.91

The bold values represent the highest accuracy and specificity.

## Data Availability

The data used to support the findings of this study are available from the corresponding author upon request. The main codes are provided in https://github.com/JZK00/Radiomics-Features-Extractor. The radiomics data are provided in https://github.com/JZK00/BM-GBM.

## Abbreviations

MRI: Magnetic resonance imaging
ROIs: Regions of interest
T1WI/T2WI: T1/T2-weighted images
T1CE: Contrast-enhanced T1
HCR: Handcrafted radiomics
DLR: Deep learning-based radiomics
ROC: Receiver operating characteristic
AUC: Area under curve
RF: Random forest
ResNet: Residual network
LoG: Laplacian of Gaussian.

## Supplementary Materials

Supplementary Materials Supplementary Materials 1: the details of original and selected features of each feature group. Supplementary Materials 2: the robust performance of the training dataset using 10-fold cross-validation.
