# Supplementary material for: Handcrafted and Deep Learning-Based Radiomic Models Can Distinguish GBM from Brain Metastasis
Source: J Oncol. 2021 Jun 3;2021:5518717. doi: 10.1155/2021/5518717 (PMC8195660; doi:10.1155/2021/5518717)

**Supplement Material 1**

**Table S1. The details of 6 feature groups.**

| Feature Group | Original features | Selected features |
| --- | --- | --- |
| T1C-HCR | 1106 | 206 |
| T1C-HCR+DLR | 2106 | 259 |
| T1WI-HCR | 1106 | 147 |
| T1WI-HCR+DLR | 2106 | 185 |
| T2WI-HCR | 1106 | 136 |
| T2WI-HCR+DLR | 2106 | 137 |

**Supplement Material 2**

**Figure S2.** The ROC curves of the 10-fold cross-validation of 6 single-modality models in training data-set. a-c were the validation of HCR models of T1C, T1WI, and T2WI, respectively. d-f were the HCR+DLR models of T1C, T1WI, and T2WI, respectively.


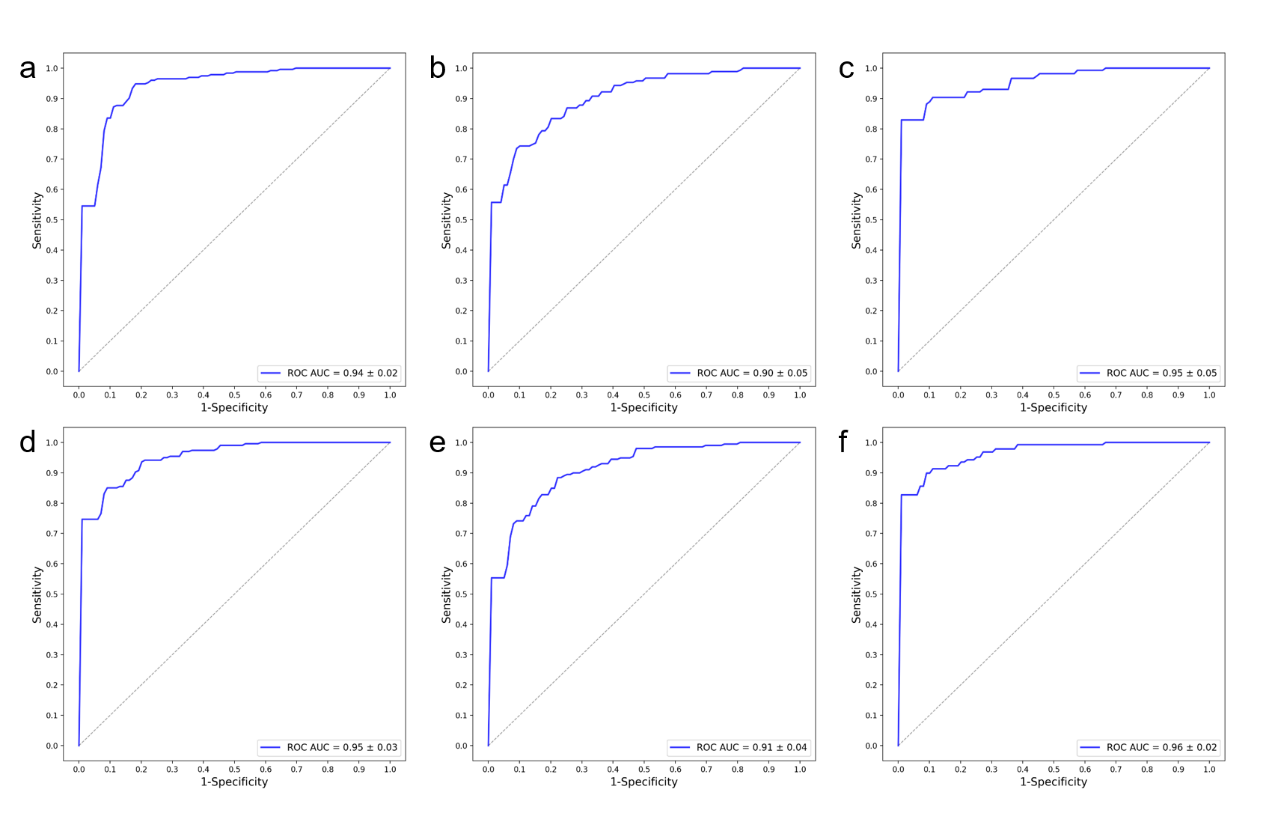

Supplement: Supplementary Materials — 1: the details of original and selected features of each feature group. Supplementary Materials 2: the robust performance of the training dataset using 10-fold cross-validation. [file 5518717.f1.docx]
